# Musculoskeletal ultrasound in symptomatic thumb-base osteoarthritis: clinical, functional, radiological and muscle strength associations

**DOI:** 10.1186/s12891-019-2610-4

**Published:** 2019-05-17

**Authors:** Win Min Oo, Leticia A. Deveza, Vicky Duong, Kai Fu, James M. Linklater, Edward A. Riordan, Sarah R. Robbins, David J. Hunter

**Affiliations:** 1 Rheumatology Department, Royal North Shore Hospital and Institute of Bone and Joint Research, Kolling Institute, University of Sydney, Sydney, Australia; 2 Department of Musculoskeletal Imaging, Castlereagh Sports Imaging Center, St. Leonards, Sydney, Australia

**Keywords:** Ultrasonography, Hand osteoarthritis, Arthritis, Inflammation

## Abstract

**Background:**

Thumb-base osteoarthritis (OA) is a common cause of pain and disability This study aimed to investigate the associations of musculoskeletal ultrasound OA pathologies with the extent of pain, function, radiographic scores, and muscle strength in symptomatic thumb-base osteoarthritis.

**Methods:**

This is a cross-sectional study of an ongoing clinical trial with eligibility criteria including thumb-base pain on Visual Analogue Scale (VAS) ≥40 (0 to 100 mm), Functional Index for Hand OA (FIHOA) ≥ 6 (0 to 30) and Kellgren Lawrence (KL) grade ≥ 2. The most symptomatic side was scanned to measure synovitis and osteophyte severity using a 0–3 semi-quantitative score, power Doppler and erosion in binary score. A linear regression model was used for associations of ultrasound findings with VAS pain, FIHOA and hand grip and pinch strength tests after adjusting for age, gender, body mass index, disease duration and KL grade as appropriate. For correlation of ultrasound features with KL grade, OARSI ((Osteoarthritis Research Society International) osteophyte and JSN scores, Eaton grades, Spearman coefficients were calculated, and a significant test defined as a *p*-value less than 0.05.

**Results:**

The study included 93 participants (mean age of 67.04 years, 78.5% females). Presence of power Doppler has a significant association with VAS pain [adjusted β coefficient = 11.29, *P* = 0.02] while other ultrasound pathologies revealed no significant associations with all clinical outcomes.

In comparison to radiograph, ultrasonographic osteophyte score was significantly associated with KL grade [r_s_ = 0.44 (*P* < 0.001)], OARSI osteophyte grade [r_s_ = 0.35 (*P* = 0.001)], OARSI JSN grade [r_s_ = 0.43 (P < 0.001)] and Eaton grade [r_s_ = 0.30 (*P* < 0.01)]. Ultrasonographic erosion was significantly related with radiographic erosion [r_s_ = − 0.49 (P = 0.001)].

**Conclusion:**

From a clinical perspective the significant relationship of power Doppler with pain severity in thumb base OA suggests this might be a useful tool in understanding pain aetiology. It is important to recognise that power Doppler activity was only detected in 14% of the study so this might be an important subgroup of persons to monitor more closely.

**Trial registration:**

Registered at Australian New Zealand Clinical Trials Registry (ANZCTR), http://www.anzctr.org.au/, ACTRN12616000353493.

## Background

Thumb-base osteoarthritis (OA) denotes structural alteration of the thumb carpometacarpal joint with a female predominance up to 6:1 [1]. It is a common cause of pain and disability, restricting the ability to perform simple tasks of daily living, and is characterized by hand weakness and radiographic abnormalities [2]. The lifetime prevalence is nearly 10%, with the epidemiological radiographic prevalence varying from 4 to 33% for middle-aged and elderly populations [3].

OA is traditionally imaged with plain radiograph which has several limitations, such as inability to visualize soft tissue pathologies which can contribute to pain and symptoms [4]. Ultrasound may afford some advantages including higher sensitivity for detecting osteophytes than plain radiographs [5, 6]. In addition, the use of ultrasound would permit the study of OA phenotypes with respect to inflammatory and structural changes that cannot be visualized with a plain radiograph [7].

A number of studies have examined the association of ultrasound findings with symptoms, function and radiographic findings in multifocal hand OA [7, 8] and other large joints such as knee and hip [9–12]; however, only three studies utilized ultrasound specifically for thumb-base OA, pinpointing on comparative prevalence of ultrasound-detected effusion (31 OA vs 37 controls) [13], the relationship of ultrasound features with disability (*n* = 57) [14] and the association of inflammatory ultrasound features with presence of pain on palpation (*n* = 87) [15]. As a diagnostic tool to be used in clinical research and practice, the validity of the tool should be determined using comparators such as disease symptoms, functional status in daily living activities, strength and other routine imaging. As yet, there is a lack of ultrasound studies focusing on its construct validity using all relevant symptomatic and structural outcomes as comparators in thumb-base OA.

This study aimed to determine the associations of ultrasound features of OA with extent of pain at the thumb-base joint, grip and pinch strength, functional score and radiographic findings.

## Method

### Study design and participant selection

This is a cross-sectional analysis from baseline assessment of the ongoing COMBO (Effect of Combined Conservative Therapies on Clinical Outcomes in Patients with Thumb-base Osteoarthritis) clinical trial starting from May 2016 (Trial registration No: ACTRN12616000353493) [16]. Approval for this study was obtained from the local research ethics committee (HREC/15/HAWKE/479).

Participants were recruited from the community and our research volunteer database by using the recruitment strategies such as affixation of posters/flyers on notice boards of waiting rooms of medical practices and community areas; advertisement in newsletters, radio, and local and major newspapers and advertisements on social media networks. Firstly, a preliminary screening was conducted by phone/internet, and then if the participant passed this initial screening, a face-to-face visit was arranged to confirm their eligibility. The inclusion criteria were: 1) age ≥ 40 years; 2) thumb-base pain at least half of the days in the past month; 3) average pain ≥40 on a 100 mm Visual Analogue Scale (VAS) [17] over the 48 h prior to the study enrollment; 4) Functional Index for Hand Osteoarthritis scores ≥6 (FIHOA, range 0–30) [18]; 5) Kellgren Lawrence grade (KLG) [19] ≥2 in the index thumb-base joint.

Exclusion criteria were: 1) known diagnosis of crystal-related arthritis (e.g., gout); 2) autoimmune arthritis (e.g., rheumatoid arthritis); 3) hemochromatosis 4) fibromyalgia; 5) significant injury to the index joint in the past 6 months; 6) any other self-reported hand condition that is likely to cause pain at the thumb base (e.g., scaphoid fracture). All participants provided informed consent.

The most symptomatic hand, as defined by pain on VAS score or worst function over the prior 48 h if the same VAS score in both hands, was included in cases of bilateral symptomatic thumb-base OA.

The cohort included here is a convenience sample recruited from the baseline visit of the COMBO clinical trial, and all participants available for an ultrasound visit between May 2016 and August 2017 were included. One hundred and seventy-two potential participants were screened to get the current sample size.

### Clinical, functional and radiological assessment

Demographic data such as age, gender, height, weight and symptom duration were collected. Pain at the thumb base was scored on a 100 mm VAS. Bilateral grip and tip-pinch strength measured in kilogram-force (Kg-F), using the hand dynamometer (Jamar Hand Dynamometer, Model: A7291, Patterson Medical) and pinch gauge (Model: PG-30, B&L Engineering), respectively. Participants were seated with both feet flat on the ground and the elbow flexed at 90 degrees and were instructed to use their maximum force; the average score of the three trials was used in the analysis.

Hand function was assessed by FIHOA questionnaire which includes ten self-reported items scored on a 4-point Likert scale of 0 (possible without difficulty) to 3 (impossible). The outcomes measures were validated instruments recommended to be measured in hand OA clinical trials [20].

Bilateral hand radiograph (posteroanterior view) was used to score KLG [19], osteophyte and joint space narrowing (JSN) scores of the Osteoarthritis Research Society International (OARSI) atlas [21], and Eaton classification [22]. Radiographic KLG, OARSI osteophyte and JSN were graded by a rheumatologist (LD), and Eaton grades by a physician (ER), respectively. The intra-rater reliability was assessed using 20 radiographs with a 6-month interval between two sessions, providing the weighted kappa of (0.76, 0.72, 0.78, and 0.82) for KLG, OARSI osteophyte, OARSI JSN and Eaton grade, respectively.

### Ultrasound examination

The physician sonographer (WMO, four years of musculoskeletal ultrasound experience, designated with a RhMSUS certification by American College of Rheumatology and having attended EULAR ultrasound courses) performed the ultrasound on the index hand in the air-conditioned radiological setting, being unaware of the other clinical and radiographic outcomes. The thumb-base joint was scanned on the longitudinal and transverse plane of the palmar and dorsal aspect according to the OMERACT ultrasound definitions and scanning methods of published papers [23, 24]. A 12 MHz linear probe (L12–4, Philips Sparq Model) was used with fixed ultrasound parameters throughout the study. Power Doppler was assessed with a frequency of 4.4 MHz and medium wall filter, using minimal pressure during the scanning. The gain was adjusted until the background signal was removed.

**Fig. 1:**
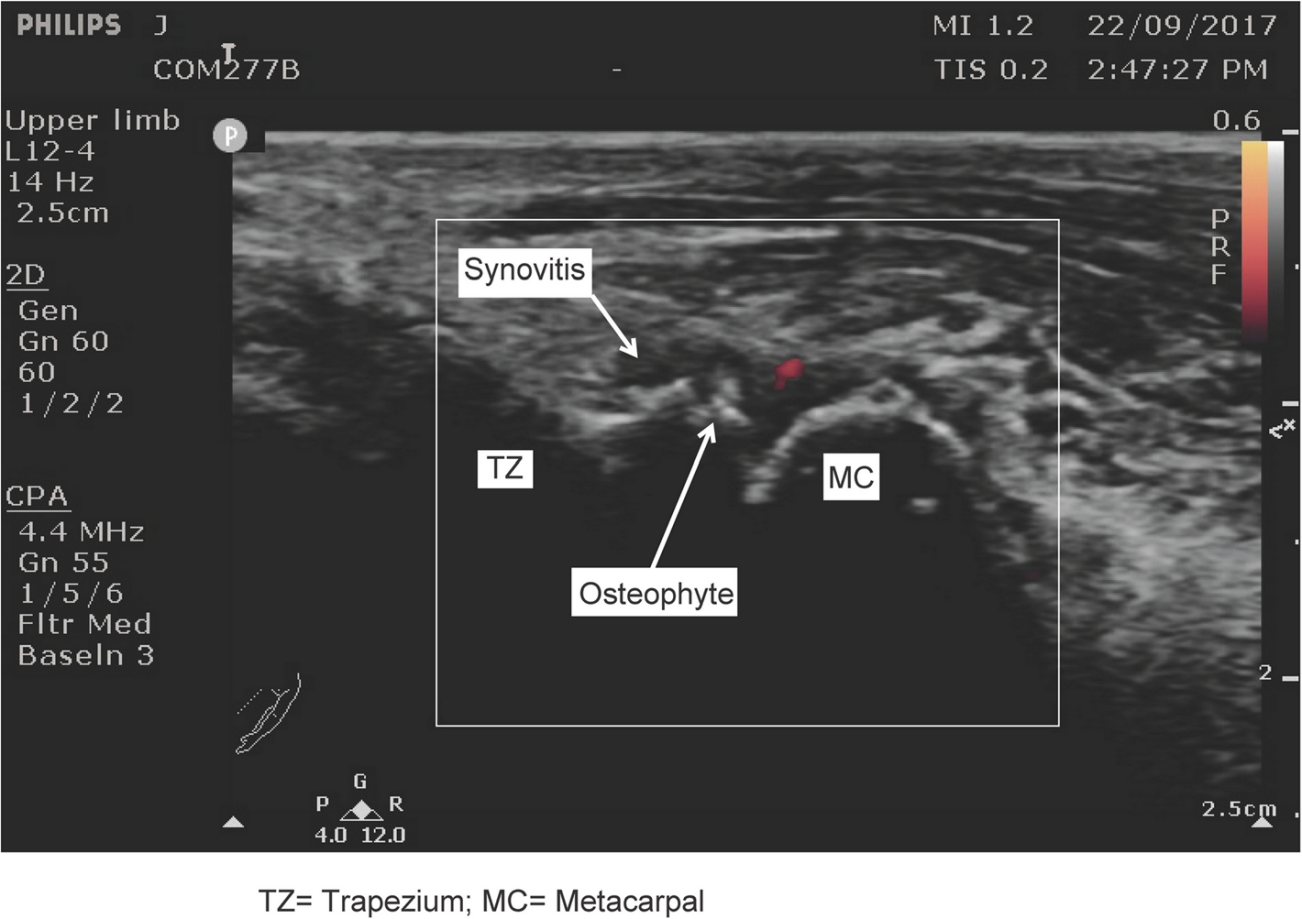
Power Doppler activity in thumb-base osteoarthritis. TZ = Trapezium; MC = Metacarpal

**Fig. 2:**
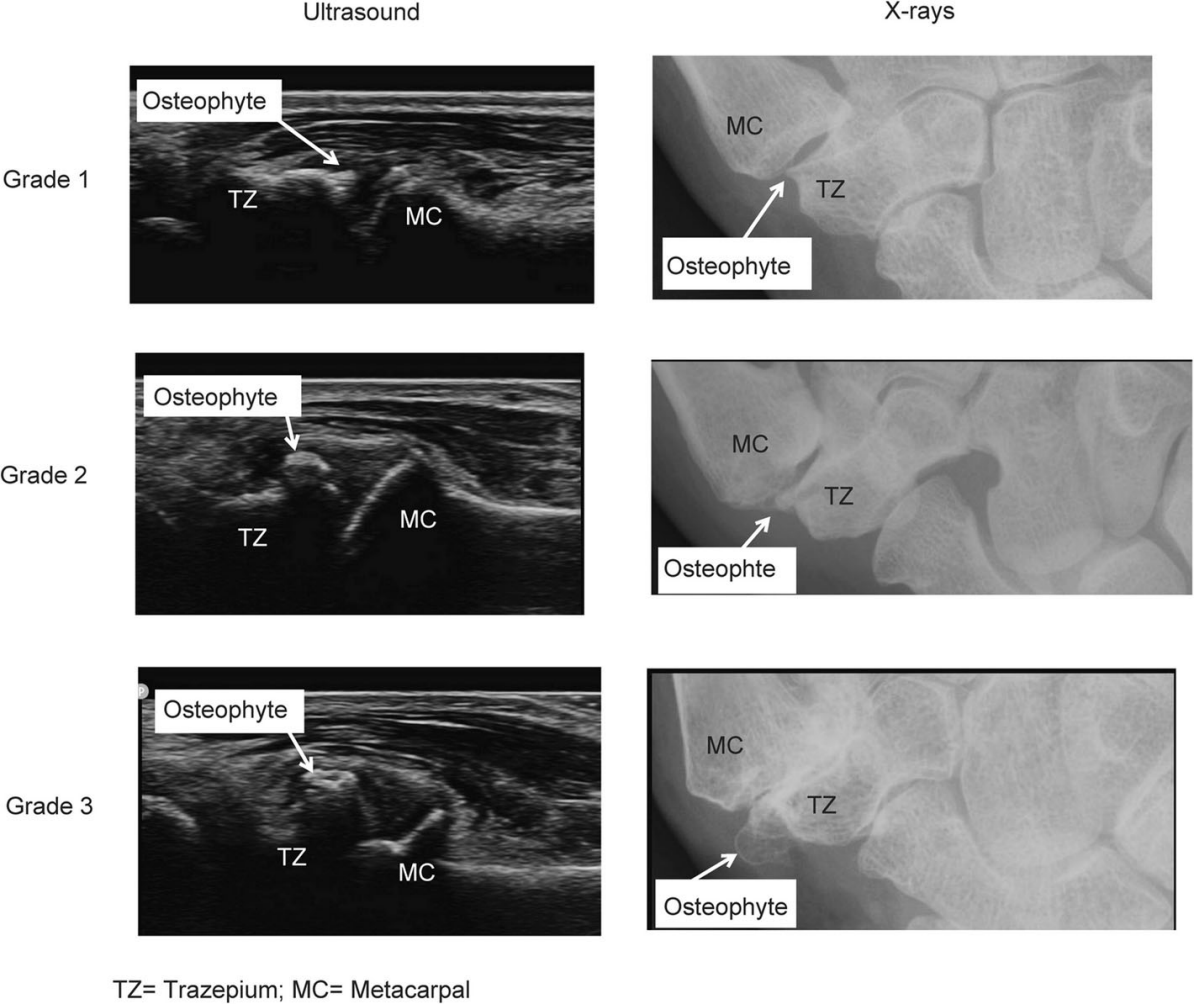
Atlas for Osteophyte grading of ultrasound and plain radiograph in our sample. Grade 1 = mild; Grade 2 = moderate; Grade 3 = severe. TZ = Trapezium; MC = metacarpal

Effusion was defined as hypoechoic or anechoic fully compressible material, synovial hypertrophy as echogenic or hypoechoic slightly compressible or non-compressible intra-articular tissue [25]. Synovial hypertrophy and effusion were considered together as a single domain “synovitis” which was graded on a 0–3 scale (absent, mild, moderate and severe) as suggested by Keen et al [24]. Doppler signal as a pulsating colour spot found within the synovial structure [23], and graded in binary score (present/absent) (Fig. 1). Osteophytes were defined as cortical protrusions at the joint margin seen in two planes [23], and severity of osteophytes was scored semi-quantitatively (0–3) using the atlas by Mathiessen et al. [26], based on the largest osteophyte independently of the number, size and location of other osteophytes (Fig. 2). Erosion was defined as an intra-articular discontinuity of the bone surface that is visible in two perpendicular planes [23] and scored on a binary scale. An evaluation sheet form was used for documenting the ultrasonographic findings.

### Intra-rater reliability

Utilizing still images of 40 randomly selected cases, the intra-rater reliability was examined 6 months after the first session, with a K_W_ value of 0.77 (0.60 to 0.94) for synovitis, 0.79 (0.63 to 0.96) for osteophyte, and unweighted kappa of 0.89 (0.69 to 1.00) for power Doppler.

### Inter-machine reliability

To evaluate the inter-machine reliability, the same scanning procedures and scoring system were performed in 40 patients, using a latest high-end ultrasound machine (Aplio Platinum 500, Toshiba, Japan) equipped with multi-frequency linear transducers (6-18 MHz). The B-mode and power Doppler settings of the machine were optimized by the application specialist from Toshiba. Due to low prevalence of some ultrasound pathologies, prevalence-adjusted bias-adjusted kappa (PABAK) was calculated, giving rise to a PABAK value of 0.81(0.65, 0.97) and percentage agreement of 87.5% for synovitis, 0.78(0.60, 0.95) and percentage agreement of 85% for osteophyte, 0.60(0.34,0.86) and percentage agreement 80% for power Doppler.

### Statistics

To investigate whether US features were independently associated with pain, function and strength tests, linear regression analyses were conducted for synovitis and power Doppler, adjusting for age, sex, body mass index (BMI), duration of disease and KLG. Adjustments for age, sex, body mass index (BMI), duration of disease were performed for regressing structural ultrasound features such as osteophyte, erosion. Spearman correlations were calculated to calculate the relationship of ultrasound features with radiographic gradings. Correlation coefficients were interpreted according to the Evans’ classification [27], <0.20:very weak; 0.20–0.39:weak; 0.40–0.59:moderate; 0.60–0.79;strong and >0.80:very strong. All statistics were conducted with SPSS version 23 and a significant association/correlation was defined as a *p*-value less than 0.05.

## Results

### Demographic and clinical characteristics

A total of 93 participants were included in this study with 73 females. The demographics of the participants are shown in Table 1.

**Table 1:** Baseline, clinical and radiographic data of study participants

Population, n	93
Age, mean (S.D.); years	67.04 ± 6.95
Female, n (%)	73 (78.5%)
BMI, mean (S.D.); kg/m2	29.35 ± 6.73
Disease duration, mean (S.D.), years	3.06 ± 1.10
VAS pain, mean (S.D.)	61.61 ± 14.37
Pinch Strength, mean (S.D.), Kg-F	3.21 ± 1.16
Grip Strength, mean (S.D.), Kg-F	20.06 ± 8.16
FIHOA, mean (S.D.)	11.33 ± 3.91
Kellgren and Lawrence grade, n (%)	
0	0
I	0
II	27 (29.0)
III	48 (51.6)
IV	18 (19.4)
OARSI osteophyte, n (%)	
0	6 (6.5)
I	37 (39.8)
II	21 (22.6)
III	29 (31.2)
OARSI JSN, n (%)	
0	13 (14.0)
I	28 (30.1)
II	33 (35.5)
III	19 (20.4)
Eaton grade, n (%)	
0	2 (2.2)
I	22 (23.7)
II	18 (19.4)
III	47 (50.5)
Radiographic erosion on X-rays, n (%)	2 (2.2)

*BMI* Body mass index, *FIHOA* Functional index for hand osteoarthritis, *JSN* Joint space narrowing, *OARSI* Osteoarthritis research society international, *VAS* Visual analogue scale

### Radiographic findings

According to KLG, grade 3 was found in more than half of the participants (*n* = 48,51.6%), grade 2 in 27 (29.0%) and grade 4 in 18 (19.4%). Osteophytes were not detected in 6 (6.5%) of participants, respectively, using the OARSI atlas. Radiographic erosion was present in 2 participants. The distribution of all radiographic findings is outlined in Table 1**.**

### Distribution of ultrasound-detected pathologies

On ultrasound, synovitis and power Doppler was detected in 52 (55.9%) and 13 (14.0%), respectively. No participants showed severe synovitis (grade 3) on ultrasound. The majority of participants (*n* = 65, 69.9%) demonstrated large osteophytes on ultrasound. Ultrasound-detected erosion was found in 2 patients. The frequency of different ultrasound findings is shown in Table 2.

**Table 2:** Ultrasonographic findings in study participants

Population, n	93
Synovitis, n (%)	
0	41 (44.1)
I	36 (38.7)
II	16 (17.2)
III	0
Power Doppler, n (%)	13 (14.0)
Osteophyte, n (%)	
0	0
I	3 (3.2)
II	25 (26.9)
III	65 (69.9)
Erosion on ultrasound, n (%)	2 (2.2)

There were significant associations synovitis vs erosion (r_s_ = 0.23 (*P* = 0.026).

### Association of ultrasound findings with pain, strength and function

The presence of power Doppler was significantly associated with degree of VAS pain [βcoefficient = 11.29, P = 0.02] after adjusting the confounders. The synovitis and osteophyte were not significantly associated with pain, pinch and grip strength, and FIHOA score (Table 3).

**Table 3:** Association between ultrasound-detected pathologies and clinical and functional measures

	Synovitis^a^	Power Doppler^a^	Osteophyte^b^	Erosion^b^
VAS pain				
Adjusted β	0.60	11.29	0.24	−12.91
(95% CI)	(−3.91–5.12)	(2.47–20.12)	(− 6.12–6.61)	(− 33.88–8.07)
P (2-tailed)	0.79	**0.02**	0.94	0.22
Pinch strength				
Adjusted β	0.120	−0.01	−0.16	0.85
(95% CI)	(−0.22–0.46)	(− 0.63–0.66)	(−0.64–0.33)	(− 0.76–2.46)
P (2-tailed)	0.48	0.97	0.53	0.30
Grip Strength				
Adjusted β	0.82	−0.71	1.27	1.84
(95% CI)	(−1.17–2.81)	(−4.56–3.13)	(− 1.50–4.04)	(−7.28–10.97)
P (2-tailed)	0.42	0.71	0.36	0.69
FIHOA				
Adjusted β	−.35	0.40	0.21	−2.84
(95% CI)	(−1.47–0.78)	(−1.93–2.72)	(−1.52–1.94)	(−8.53–2.86)
P (2-tailed)	0.54	0.74	0.81	0.32

*Β* β coefficient, *FIHOA* Functional index for hand osteoarthritis, *VAS* Visual analogue scale;

95% CI = 95% confidence interval

^a^Adjusted for age, sex, and body mass index, disease duration and KL grade

^b^Adjusted for age, sex, body mass index, and disease duration

### Association of ultrasound findings with radiographic findings

The ultrasonographic osteophyte scores were significantly correlated with KLG [r_s_ = 0.44 (*P* < 0.001)], OARSI osteophyte grade [r_s_ = 0.35 (*P* = 0.001)], OARSI JSN grade [r_s_ = 0.43 (P < 0.001)] and Eaton grade [r_s_ = 0.30 (*P* < 0.01)] as shown in Table 4. Erosion detected on ultrasound had a correlation of 0.49 with radiographic erosion as ultrasound could not visualize the radiographic erosion in one patient with florid osteophytes. In addition, in 6 patients, ultrasound could detect osteophytes which the plain radiograph could not.

**Table 4:** Relationship between ultrasound-detected pathologies and radiological findings

	Synovitis	Power Doppler	Osteophyte	Erosion
KL score				
r_s_	−0.09	− 0.03	0.44	− 0.09
P (2-tailed)	0.41	0.76	0.001	0.41
OARSI OST				
r_s_	−0.13	−0.14	0.35	−0.13
P (2-tailed)	0.21	0.19	0.001	0.22
OARSI JSN				
r_s_	−0.03	−0.06	0.43	−0.08
P (2-tailed)	0.75	0.57	0.001	0.43
Eaton SUB				
r_s_	−0.11	−0.01	0.30	−0.03
P (2-tailed)	0.29	0.98	0.01	0.75
Erosion				
r_s_	0.15	0.15	0.10	0.49
P (2-tailed)	0.14	0.14	0.36	0.001

*KL* Kellgren Lawrence, *OARSI* Osteoarthritis research society international; *OST* Osteophyte, *rs* Spearman’s correlation, *SUB* Subluxation

## Discussion

The current study revealed the frequent finding of some ultrasound pathologies, the significant association of the presence of power Doppler with the severity of pain, and significant correlations of ultrasound-detected osteophyte with radiographic scores in thumb-base OA. However, the study could not detect any significant correlation of ultrasound pathologies with strength and functional measures.

This study showed that synovitis, when present, were mostly scored toward the lower end of the semi-quantitative scale as these grading scores were adopted from the scoring system created originally for rheumatoid arthritis [23], which is quantitatively different in inflammatory severity from OA [28]. Recent papers questioned the use or relevance of semi-quantitative scores in OA as it can lead to unequal distribution of the scores [29] and floor effects causing less sensitivity to detect an improvement in interventional trials [30].

Our participants had worse grades of osteophyte compared to the counterparts of thumb-base joint recorded in multifocal hand OA study by Naguib et al. [8]. This discordant result might be accounted for by the older age in our study population and different study selection criteria (American College of Rheumatology criteria vs radiological criteria), number of joint involvement (multifocal vs mono-articular OA) and severity of the disease. Structural changes of the hand joints tend to be more commonly found with increasing age. About 6% of adults aged > 30 years [31] and 13% of persons aged 60 and over [32] had radiographic OA features. Such demographic and selection criteria differences might lead to our study population having more participants with fully established OA features.

Poor correlation between clinical symptoms and radiographic findings has previously been demonstrated in knee OA [33], and a similar discordance was suggested by our findings which revealed significant association of only power Doppler with VAS pain, and no significant association with other ultrasound features. The finding of a significant correlation of power Doppler signal is in agreement with increasing evidence of MRI literature, which implied that active synovial inflammation plays a critical role as pain generator of OA [34, 35]. This result is also consistent with meta-analytic reports in knee OA ultrasound [30].

However, the lack of significant correlation of grey-scale synovitis with pain raised several questions about its role in pain generation in OA. Hall et al. [36] postulated that perhaps synovial hypertrophy as seen on grey-scale ultrasound might not be inflammatory as grey-scale ultrasound cannot differentiate between active and indolent synovitis, tissue debris and fibrosis. Synovial hypertrophy and effusion could be the results of altered joint biomechanics [37] and reduction in lymphatic vessels [38]. In addition, pain in OA can be partly due to bone marrow oedema (BMOs) [39], which ultrasound cannot detect as sound waves cannot penetrate the bone, reducing the strength of correlation between grey-scale synovitis and VAS pain. The other reason might be a measurement issue. Pain is a subjective phenomenon, and inter-individual differences may modify the pain experience and intensity [40]. Subjects sustaining the same degree of structural damage experienced widely different degrees of pain, a phenomenon that is poorly elucidated [41]. Kroon et al reported no significant association between inflammatory OA features of ultrasound and presence of pain on palpation although MRI synovitis and BMOs showed a significant relationship with pain in a different cohort [15]. In multifocal hand OA as well, conflicting results were reported in this aspect as Keen et al. [7] reported no significant association of synovitis, power Doppler, osteophyte and joint space width (JSW) with pain whilst Naguib et al. [8] documented a significant relationship of osteophyte, JSW and cartilage thinning with pain.

The relationship of grip and pinch strength with OA imaging features are broadly discordant in the radiological literature [42]. We found no correlation between ultrasound features and grip or pinch strength, which was contradictory with those of Naguib et al. [8], which found that significant associations existed between the grip strength and osteophyte in multifocal hand OA (*n* = 30). However, Naguib et al. [8] did not find a significant correlation between strength and JSW, which was comparable with our findings. This disparity might be perhaps due to demographic differences such as greater strength (19.3 Kg-F vs 15.0 Kg-F) and older age (67.3 vs 60.0 years) in our study. Baron et al. [43] did not find a correlation between hand function, grip strength, and radiographic features of hand OA, and postulated that hand function and strength were related more to neuromuscular condition than to the articular damage.

Regarding the correlation between ultrasound features and functional measures, the current study was consistent with most of the multifocal hand OA reports in the literature [7, 14, 44]. In multifocal hand OA, Keen et al. [7] demonstrated that synovitis, power Doppler and osteophyte had no significant correlation with functional impairments, utilizing the Australian/Canadian Osteoarthritis Hand Index (AUSCAN) while Koutroumpas et al. [44] reported no correlation of synovitis and power Doppler with FIHOA score. In thumb-base OA, most ultrasound features had no correlation with Disabilities of the Arm Shoulder and Hand (DASH) score [14]; the only difference being that they found a correlation of osteophyte with function while we did not. However, contrary to these findings, Naguib et al. [8] determined a significant correlation of the structural features of ultrasound such as osteophyte with AUSCAN questionnaire in multifocal hand OA. It should be noted that the measures of hand function depend on multiple joints acting in concert, whereas our study looked at only one of those joints and so we could not exclude the impact of other finger joints OA on the associations. A recent meta-analysis in clinimetrics of ultrasound in knee OA reported that functional impairments are significantly but weakly correlated with effusion [r = 0.23 (0.08, 0.37)] and osteophyte [r = 0.18 (0.04, 0.31)] [30]. The reason for this discrepancy was unclear.

Our study found that ultrasound had the ability to detect osteophytes which plain radiographs failed to visualize. These findings are in agreement with those of Mathiessen et al. [26], Keen et al. [5] and Vlychou et al. [6], which demonstrated more osteophytes on ultrasound than on plain radiograph in multifocal hand OA. This can be explained by the capability of ultrasound to perform dynamic multiplanar imaging both longitudinally and transversely, and two-dimensional nature of plain radiograph which is likely to miss the small osteophyte localized to either palmar or dorsal aspect of the joint on standard PA view. However, the current radiographs are single-view only and this may position radiography at a disadvantage.

Although Vlychou et al. [6] reported that ultrasound could reveal more erosions than plain radiograph in erosive multifocal hand OA, our study could not detect more erosions on ultrasound than plain radiograph perhaps due to higher prevalence of osteophyte (100% vs 41%) and reduced number of erosive OA (2% vs 100%) in our study. In one patient, erosion was near the central joint area with the overhanging osteophyte, which could not be visualized on ultrasound due to limited acoustic window. Our finding was consistent with Keen et al. [5] who reported 6 erosions on plain radiograph (3 DIP, 2 PIP and 1 MCP); 2 joints were normal on ultrasound while the other 4 had marked osteophytosis. The similar conclusion was documented in another study [45] which implied that ultrasound could not detect 27.3% of erosions seen on plain radiograph. In small joints having severe osteophytes, deformities and subluxation, ultrasound was distinctly cumbersome due to acoustic artefacts and small acoustic window. Ultrasound appears to be more useful for detection of non-radiographic phase of erosive OA before the appearance of frank erosion which plain radiograph can visualize at this stage.

Naguib et al. [8] demonstrated the significant correlation of osteophyte with KLG, which is concordant with the current study. However, the correlation is just moderate probably due to different measurement methods of plain radiograph and ultrasound in scoring the grades of severity (each grades of ultrasound osteophyte atlas was not standardized exactly with the same grade of OARSI radiographic atlas; this might lead to over- or under-estimation of ultrasound severity score), more scanning planes for ultrasound and the fact that the comparison was not site-specific.

### Limitation

As this was a cross-sectional study, we cannot establish a cause-effect relationship and determine clinical importance of variability of the power Doppler with longitudinal changes in pain. Another limitation was the lack of a reference method such as MRI in detecting synovial and bony pathologies, and so we are not able to comment on the percentage of false positive and false negative ultrasound features. Ideally, the inter-rater reliability data should be conducted but only one ultrasound operator was available for this study. In addition, the ultrasound machine used in our study is not the optimal high-end machine equipped with the latest high-frequency probe. In an ideal world, we would also have included a cohort of healthy individuals for comparison of ultrasound pathologies. Another important study limitation was that the ultrasound operator was not blinded to diagnosis; however, in practice, blinding a sonographer to joint deformities and joint tenderness is not feasible.

## Conclusion

From a clinical perspective, the significant association of power Doppler with pain severity in thumb base OA suggests that ultrasound might be a useful tool in understanding pain aetiology. It is important to recognise that power Doppler activity was only detected in 14% of the study so this might be an important subgroup of persons to monitor more closely. In addition, the lack of association of other ultrasound structural features with hand function and strength reinforces the complex biopsychosocial origins of pain and function and the ongoing challenge of pain and structure dissociation in osteoarthritis. Further study with longitudinal follow-up may contribute to more clarification.

## Abbreviations

BMOs: Bone Marrow Oedema
FIHOA: Functional Index for Hand OA
JSW: Joint space width
KLG: Kellgren Lawrence Grading
OA: Osteoarthritis
OARSI: Osteoarthritis Research Society International
VAS: Visual Analogue Scale
